# The Resurgence of Dorsal Plating for Distal Radius Fractures in Malaysia: An Epidemiological Study at a Tertiary Hand Centre

**DOI:** 10.7759/cureus.107949

**Published:** 2026-04-29

**Authors:** Zareze Abd Rahman, Syahril R Arsad, Rashdeen Fazwi Muhammad Nawawi

**Affiliations:** 1 Orthopaedics and Traumatology Department, Hospital Kuala Lumpur, Kuala Lumpur, MYS; 2 Hand and Microsurgery Unit, Orthopaedics Department, Hospital Selayang, Kuala Lumpur, MYS

**Keywords:** bimodal distribution, epidemiology, fragility fracture, hand surgery, malaysia, s: distal radius fracture

## Abstract

Background: This study aimed to define the contemporary epidemiological profile of adult distal radius fractures (DRFs) at a dedicated Hand and Microsurgery Centre in Malaysia.

Methods: A retrospective cross-sectional study included 387 adult patients with nonpathological DRFs presenting to the Hospital Selayang, Malaysia, over a 24-month period (January 2023-December 2024). Demographic variables, injury mechanisms, fracture classifications (OTA and Frykman), and treatment modalities were analysed. Between-gender comparisons were performed using Mann-Whitney and chi-square tests.

Results: The mean age was 52.8 ± 19.8 years, with a bimodal distribution peaking at 30-40 years and 65-75 years. Males predominated overall (n = 208, 53.7%), but with a striking age disparity: median age for females was 63 years compared to 39 years for males (p < 0.001). Fall on an outstretched hand (FOOSH) was the predominant mechanism (n = 233, 60.3%). Notably, fragility fractures constituted nearly half of all DRFs (n = 176, 45.5%), occurring predominantly in elderly females (n = 128, 72.7%) with a mean age of 70.1 ± 9.4 years. Conservative management with casting was employed in 75.7% (n = 293) of patients. Among surgically treated cases, volar rim plating (n = 27, 7%) and variable-angle locking compression plating (n = 21, 5.4%) were the most common.

Discussion: This study confirms a bimodal age distribution for DRFs in Malaysia but reveals an alarmingly high proportion of fragility fractures (n = 176, 45.5%), exceeding rates reported in Western populations. These findings mandate urgent implementation of fracture liaison services to address the secondary prevention gap. The predominance of conservative management, even for complex fractures, reflects regional practice patterns that warrant prospective evaluation against functional outcomes.

## Introduction

Distal radius fractures (DRFs) represent one of the most common upper extremity injuries encountered in orthopaedic and hand surgery practice worldwide, accounting for approximately one-sixth of all fractures treated in emergency departments [1,2]. The epidemiological profile of these fractures is characterised by a well-documented bimodal age distribution, with distinct peaks observed in younger individuals following high-energy trauma and in older adults, particularly those with osteoporotic bone, after low-energy falls [1,2]. While this bimodal pattern has been extensively described in Western populations, there is growing recognition that the epidemiology of DRFs may vary significantly across different geographic regions and ethnic groups due to differences in lifestyle, occupational risks, bone health, and trauma mechanisms.

In Asia, including Malaysia, the demographic and injury characteristics of patients sustaining DRFs may differ from those reported in Western literature. Studies from South Asian populations have demonstrated a younger mean age at presentation and a male predominance, often attributed to a higher proportion of high-energy injuries in these cohorts [1]. Within the Malaysian context, limited data exist regarding the detailed epidemiology of DRFs. A previous study from an urban tertiary medical centre in Malaysia reported a mean age of 51.3 years among 110 patients with distal radial fractures, with a slight female predominance, and identified that increasing age, female gender, and complex fracture patterns were associated with poorer functional outcomes [3].

Despite these emerging data, there remains a significant gap in the literature regarding the comprehensive epidemiological profile of DRFs in Malaysia, particularly from a dedicated hand and microsurgery centre. The absence of a formal national fracture registry in the country necessitates continued hospital-based studies to characterise fracture patterns, treatment trends, and associated demographic factors. Specifically, it remains unclear whether the classic bimodal age distribution observed in Western populations applies to the Malaysian population presenting to such a specialised centre. Previous studies failed to demonstrate the bimodal distribution [1].

Therefore, this study tested two hypotheses: (i) that DRFs in a Malaysian tertiary hand centre would demonstrate a bimodal age distribution similar to Western populations and (ii) that the proportion of fragility fractures would be comparable to published regional benchmarks. Secondary objectives included describing treatment patterns and identifying demographic factors associated with operative versus conservative management. By providing robust baseline epidemiological data, this study aims to inform preventive strategies, optimise resource allocation, and facilitate the development of standardised treatment protocols for DRFs in the Malaysian healthcare setting.

## Materials and methods

Study design and setting

A retrospective cohort design was employed to investigate all nonpathological DRFs presenting to the Hand and Microsurgery Unit of Hospital Selayang, Batu Caves, Malaysia, a tertiary referral centre. The study captured every patient diagnosed with a DRF over a consecutive 24‑month period from 1 January 2023 to 31 December 2024.

Participants and case definition

Fractures were defined as involving the distal 3 cm of the radius. The definition intentionally included open fractures, bilateral injuries, and those occurring alongside other musculoskeletal trauma to ensure comprehensive case capture. Pathological fractures secondary to primary bone tumours or metastatic disease were excluded. All patients aged ≥18 years with a confirmed DRF during the study period were included. Patients with incomplete medical records (missing key demographic or treatment data) and those aged <18 years were excluded.

Case ascertainment

Potential cases were identified from the emergency department database using the International Classification of Diseases, Tenth Revision, Clinical Modification (ICD‑10‑CM) codes S52.50, S52.51, S52.60, and S52.61 [4]. Each identified record was then manually verified by the two orthopaedic specialists against the original medical records and radiology reports to confirm the diagnosis and exclude pathological fractures. Fractures were classified according to the Orthopaedic Trauma Association (OTA) and Frykman systems by two independent reviewers: a hand surgery fellow and a consultant hand surgeon. Classification was performed using standard anteroposterior and lateral digital radiographs obtained at presentation. In cases of disagreement (n = 14, 3.6%), consensus was reached through discussion. A fragility fracture was defined as a fracture resulting from a low-energy fall (from standing height or less) in a patient aged ≥50 years, determined retrospectively from the medical record. Age < 50 years and low-energy fractures were not considered fragility fractures.

Data collection

From the medical records, a wide array of demographic and clinical variables was systematically extracted using a standardised data abstraction form. Name, identification number, and hospital registration number were de-identified and not included in the study. Variables included the following: demographics, including age, gender, and ethnicity; injury characteristics, including mechanism of injury (e.g., low-energy fall from standing height, high-energy trauma such as motor vehicle accident or fall from height), type of fracture (closed vs. open), presence of an associated ulnar styloid fracture, and presence of a fragility fracture (defined as a fracture occurring after a fall from standing height or less in patients aged ≥50 years); classification systems, such as the OTA classification and Frykman classification, as documented by the attending orthopaedic surgeon or radiologist; and treatment, which was dichotomised as conservative when no surgical procedure occurred within 21 days of diagnosis (e.g., cast immobilisation) or operative when any surgical intervention was performed within the same 21‑day window [1]. Operative cases were further stratified into external fixation, plate fixation, Kirschner wire (K‑wire) fixation, or screw fixation. All data were recorded in a standardised data collection form.

Data management and missing data

A total of 448 DRFs were recorded in the emergency department during the study period. Of these, 29 records were excluded due to incomplete data, and 32 were excluded because they were in the paediatric age group (<18 years). No imputation was performed for missing data; only complete cases were analysed.

Ethical considerations

This study was approved by the National Ethical Committee (NMRR:22-01245). The institutional ethics committee granted a waiver of informed consent, given the retrospective, observational nature of the study, and all data were anonymised prior to analysis.

Statistical analysis

Statistical analysis was conducted using IBM SPSS Statistics for Windows, Version 29 (Released 2022; IBM Corp., Armonk, New York, United States). Categorical variables are reported as frequencies with percentages, while continuous variables are summarised as mean ± standard deviation (SD) or median with interquartile range (IQR) where appropriate. Normality of continuous data was assessed using the Shapiro-Wilk test. For between-group comparisons, given the nonnormal distribution of age and other continuous variables, between‑gender comparisons were evaluated using the Mann-Whitney U test. For categorical variables, comparisons between groups, male vs. female, were performed using the chi‑square test.

For the assessment of bimodal distribution, to statistically confirm the visually apparent bimodal age distribution observed on the histogram, a TwoStep cluster analysis was performed using age as the sole continuous variable. The number of clusters was fixed at two, as the study hypothesis specifically sought to test for bimodality. Cluster quality was assessed using the average silhouette measure of cohesion and separation, where values >0.5 indicate moderate separation, >0.7 indicate strong separation, and <0.25 suggest no meaningful cluster structure. A silhouette value exceeding 0.5 was considered supportive of a bimodal distribution. For the fragility fracture subgroup, descriptive statistics were calculated separately to characterise this population. No imputation was performed for missing data; only complete cases were analysed. Statistical significance was defined as a two‑tailed p-value below 0.05. The average silhouette value was 0.5, which meets the conventional threshold for acceptable cluster structure (≥0.5). Therefore, interpret the age distribution as consistent with a bimodal pattern, acknowledging moderate overlap between the two age groups.

## Results

**Figure 1:**
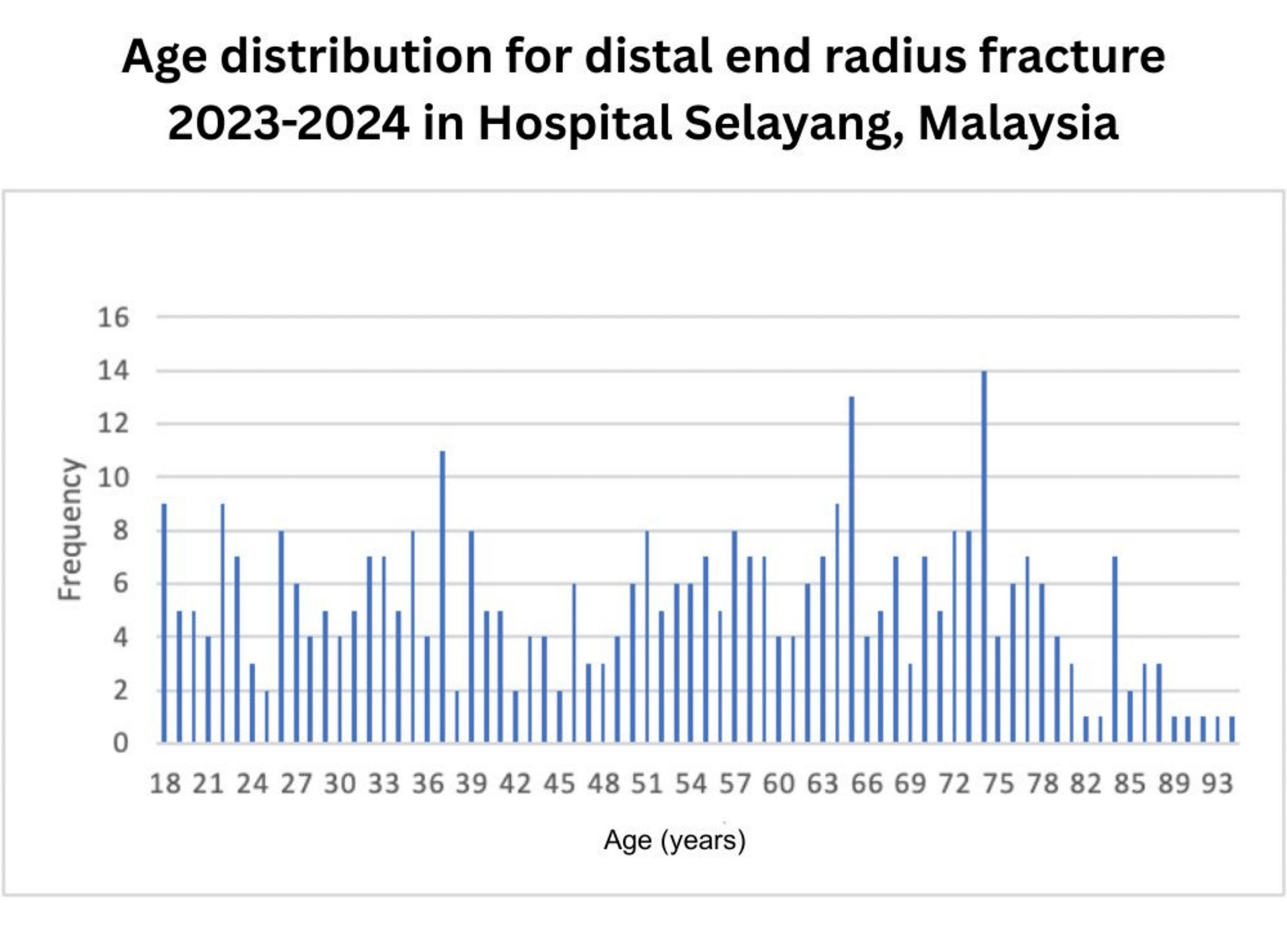
Age distribution for distal end radius fracture for the year 2023-2024 in Hospital Selayang, Malaysia Bar chart showed age-specific distribution, which peaked at 30-40 years and, again, at 65-75 years

**Figure 2:**
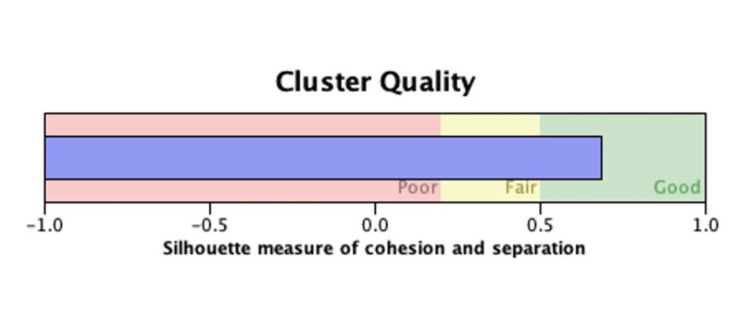
TwoStep cluster analysis Cluster quality was assessed using the average silhouette measure of cohesion and separation, showing a value of >0.5

A total of 387 patients were included in this study. The mean age was 52.8 ± 19.8 years, with a bimodal distribution (Table 1). Age-specific incidence peaked at 30-40 years and again at 65-75 years (Figure 1), which is confirmed using the average silhouette measure of cohesion and separation of >0.5 (Figure 2), indicating a bimodal distribution.

**Table 1:** Demographic data of distal end radius fracture for the year 2023-2024 in Hospital Selayang OTA: Orthopaedic Trauma Association; FOOSH: fall on an outstretched hand; MVA: motor vehicle accidents; N/A: not applicable * represents the mean (standard deviation); ^ represents the frequency (percentage); t represents the median (interquartile range); § Mann-Whitney test was used to compare between two groups, and a p-value of <0.05 is considered significant, which is the value of test statistic using a z-score. ¨ Chi-square test was used to compare between two groups, and a p-value < 0.05 is considered significant, which is the value of the test statistic using the Pearson Chi-square

Demographic	Total	Male (n = 208)	Female (n = 179)	p-value	Value of test statistic
Age	52.8 (19.8)*	39 (30) t	63 (22) t	<0.001§	-8.1
Sex					
Female	179 (46.3)^	N/A	N/A	N/A	N/A
Male	208 (53.7)^	N/A	N/A		
Mechanism of injury					
Assault	1 (0.3)^	1 (0.5)^	0 (0)^	<0.001¨	28.75
Crush injury	2 (0.5)^	1 (0.5)^	1 (0.6)^		
Fall from height	28 (7.2)^	18 (8.7)^	10 (5.6)^		
FOOSH	233 (60.2)^	100 (48)^	133 (74.3)^		
MVA	123 (31.8)^	88 (42.3)^	35 (19.6)^		
Type of fracture					
Closed fracture	365 (94.3)^	195 (93.7)^	170 (95)^	0.605¨	0.268
Open fracture	22 (5.7)^	13 (6.3)^	9 (5)^		
OTA classification					
A	273 (70.5)^	127 (61)^	146 (81.6)^	<0.001¨	19.93
B	88 (22.7)^	61 (29.3)^	27 (15.1)^		
C	26 (6.7)^	20 (9.7)^	6 (3.3)^		
Frykman classification					
1	180 (46.5)^	82 (39.4) ^	98 (54.7) ^	0.015¨	26.36
2	88 (22.7)^	45 (21.6) ^	43 (24)^		
3	63 (16.3)^	43 (20.6) ^	20 (11.2)^		
4	46 (11.9)^	34 (16.5) ^	12 (6.7)^		
5	3 (0.8) ^	0 (0)^	3 (1.7)^		
6	2 (0.5)^	0 (0)^	2 (1.1)^		
7	2 (0.5)^	1 (0.5)^	1 (0.6)^		
8	3 (0.8)^	3 (1.4)^	0 (0)^		

Males predominated overall (n = 208, 53.7%) compared to females (n = 179, 46.3%) (Table 1). Notably, the median age of female patients was 63 ± 22 years, whereas that of male patients was considerably younger at 39 ± 30 years. The most common mechanism of injury was a fall on an outstretched hand (FOOSH) (n = 233, 60.2%), followed by motor vehicle accidents (n = 123, 31.8%). The majority of fractures were closed (n = 365, 94.3%), with open fractures accounting for 5.7% (n = 22). According to the Frykman classification, the most frequent fracture types were Frykman I (n = 180, 46.5%), Frykman II (n = 88, 22.7%), Frykman III (n = 63, 16.3%), and Frykman IV (n = 46, 11.9%). According to the Arbeitsgemeinschaft für Osteosynthesefragen (AO)/OTA classification, the most frequent type was OTA A (n = 273, 70.5%), OTA B (n = 88, 22.7%), and OTA C (n = 26, 6.7%). The median age for OTA A, B, and C was 58 ± 33.5 years, 43.5 ± 29.7 years, and 31 ± 25 years, respectively. The median age for Frykman I and II fractures was 58 ± 30.7 years and 59 ± 35 years, respectively, whereas Frykman III fractures occurred at a younger median age of 37 ± 30 years. The most commonly associated fracture involved the ulnar (n = 19, 4.9%).

Regarding management, 75.7% (n = 293) of patients received conservative treatment with an above-elbow plaster of Paris (POP) cast. Among surgically treated cases, volar rim plating was the most frequently employed technique (n = 27, 7%), followed by variable angle locking compression plating (VALCP) (n = 21, 5.4%), dynamic compression plating (n = 12, 3.1%), volar rim plating with dorsal plating (n = 13, 3.3%), K-wiring (n = 10, 2.6%), external fixation (n = 7, 1.8%), screw fixation (n = 2, 0.5%), the Sauvé-Kapandji procedure (n = 1, 0.3%), and dorsal plating (n = 1, 0.3%). For OTA A fractures, above-elbow POP was used in 88.6 % (n = 242), followed by dynamic compression plating (4%, n = 11), and VALCP (1.1%, n = 3). For OTA B fractures, conservative management was also predominant (55.7%, n = 49), with volar rim plating (28.4%, n = 25) and volar rim plating with dorsal plating (14.8%, n = 13) being the most common surgical options. On the other hand, in OTA C fractures, above-elbow POP accounted for 7.7% (n = 2), and VALCP 69.2% (n = 18) is the most common procedure performed. All were listed in Table 2.

**Table 2:** Type of management for distal end radius fracture for the year 2023-2024 in Hospital Selayang VALCP: variable angle locking compressing plate; OTA: Orthopaedic Trauma Association ^ represents frequency (percentage), significant Chi-square test was used to compare between two groups, and a p-value < 0.05 is considered significant, which is the value of the test statistic using Pearson chi-square

Demographic	Above elbow pop	Dorsal plating	Dynamic compression plate	External fixation	Headless screw fixation	K-wire	Sauvé-Kapandji	VALCP	Volar locking plate and dorsal plating	Volar rim plate	Volar rim plate with dorsal plate	Total	p-value	Value of test statistic
Sex														
Female	148 (82.7)^	1 (0.6) ^	5 (2.8) ^	3 (1.7) ^	0 (0) ^	4 (2.2)^	0 (0) ^	6 (3.4) ^	1 (0.6) ^	6 (3.4)^	5 (2.8)^	179 (100)^	0.126	15.1
Male	145 (69.7) ^	0 (0) ^	7 (3.3) ^	4 (1.9) ^	2 (1) ^	6 (2.9)^	1 (0.5) ^	15 (7.2)^	1 (0.5) ^	21 (10.1)^	6 (2.9)^	208 (100)^		
OTA classification														
A	242 (88.6) ^	0 (0) ^	11 (4) ^	5 (1.8) ^	1 (0.4) ^	10 (3.7) ^	0 (0) ^	3 (1.1) ^	0 (0) ^	1(0.4)^	0 (0) ^	273 (100)^	< 0.001	393.1
B	49 (55.7)^	1 (1.1) ^	0 (0) ^	0 (0) ^	0 (0) ^	0 (0) ^	0 (0) ^	0 (0) ^	2 (2.3) ^	25 (28.4)^	11 (12.5) ^	88 (100)^		
C	2 (7.7) ^	0 (0) ^	1 (3.8) ^	2 (7.7) ^	1 (3.8) ^	0 (0) ^	1 (3.8) ^	18 (69.2)^	0 (0) ^	1 (3.8)^	0 (0) ^	26 (100)^		

A total of 176 fractures (45.5%) were classified as fragility fractures, with a mean age of 70.1 ± 9.4 years (Table 3). This subgroup was predominantly female (n = 128, 72.7%) compared to male (n = 48, 27.3%). All injuries in this group resulted from a FOOSH (100%), and 97.7% (n = 172) were closed fractures. The most common OTA types among fragility fractures were OTA A (n = 150, 85.2%), B (n = 24, 13.6%), and C (n = 2, 1.1%). Remarkably, 95.5% (n = 168) of fragility fractures were managed conservatively.

**Table 3:** Demographic data of fragility fracture in distal end radius fracture for the year 2023-2024 in Hospital Selayang OTA: Orthopaedic Trauma Association; FOOSH: fall on an outstretched hand; MVA: motor vehicle accident; N/A: not applicable * represents mean (standard deviation); ^ represents frequency (percentage); t represents median (interquartile range) § The Mann-Whitney test was used to compare two groups, and a p-value < 0.05 is considered significant, which is the value of the test statistic using the z-score. ¨ Chi-square test was used to compare two groups, and a p-value < 0.05 is considered significant, which is the value of the test statistic using the Pearson Chi-square

Demographic	Total	Male (n = 48)	Female (n = 128)	p-value	Value of test statistic
Age	70.1 (9.4)*	71 (12)t	70 (14) t	0.108§	2558.5
Sex					
Female	128 (72.7)^	N/A	N/A	N/A	N/A
Male	48 (27.3)^	N/A	N/A		
Mechanism of injury					
FOOSH	176 (100)^	N/A			
OTA classification					
A	150 (85.2)^	38 (79.2)^	112 (87.5)^	0.355¨	2.07
B	24 (13.6)^	9 (18.8)^	15 (11.7)^		
C	2 (1.1)^	1 (2.1)^	1 (0.8)^		
Frykman classification	N/A	N/A	N/A	N/A	N/A
1	97 (55.1)^	23 (47.9)^	74 (57.8)^	0.313¨	5.993
2	50 (28.4)^	15 (31.3)^	35 (27.3)^		
3	13 (7.4)^	4 (8.3)^	9 (7.0)^		
4	12 (6.8)^	5 (10.4)^	7 (5.5)^		
5	3 (1.7)^	0 (0)^	3 (2.3)^		
6	0 (0)^	0 (0)^	0 (0)^		
7	0 (0)^	0 (0)^	0 (0)^		
8	1 (0.6)^	1 (2.1)^	0 (0)^		

## Discussion

This study presents a contemporary epidemiological analysis of 387 DRFs managed at a dedicated Hand and Microsurgery Centre in Malaysia. Our findings confirm a bimodal age distribution, document a substantial burden of fragility fractures, and reveal evolving surgical practices, including the increasing use of dorsal plating techniques. 

The bimodal age distribution of DRFs is a well-established epidemiological phenomenon in Western populations, with characteristic peaks in young adults following high-energy trauma and in elderly individuals, particularly women, after low-energy falls [3,5]. Our study clearly demonstrates two distinct age peaks at 30-40 years and 65-75 years, with an overall mean age of 52.8 ± 19.8 years. A South Asian series from India reported a younger mean age of 43.2 years with male predominance, attributing this to a high proportion of motorcycle-related injuries [6].In contrast, a large Japanese population-based study confirmed a classical bimodal pattern with peaks in young males and elderly females, similar to Western data [7]. Our findings align more closely with the Japanese experience, suggesting that in well-developed Asian trauma systems serving ageing populations, the bimodal distribution becomes increasingly apparent.

The sex-specific age disparity in our cohort is particularly instructive. Female patients had a median age of 63 ± 22 years, while male patients were considerably younger at 39 ± 30 years. This pattern is consistent with data from the National Trauma Data Bank in the United States, where male patients with DRFs were significantly younger than their female counterparts [8]. Diamantopoulos et al. similarly reported that high-energy DRFs in younger males and low-energy fragility fractures in older females represent two distinct epidemiological entities requiring different preventive approaches [9].

Perhaps the most clinically significant finding of our study is that 45.5% of all DRFs were classified as fragility fractures, occurring predominantly in elderly females (72.7%) with a mean age of 70.1 ± 9.4 years following a simple FOOSH. This proportion is substantially higher than that reported in some Western series. For instance, Candela et al. found that fragility fractures accounted for approximately 30% of their cohort in a suburban Italian population [10]. The higher proportion in our study may reflect both an ageing Malaysian population and potential referral bias to a tertiary hand surgery centre.

The DRF is increasingly recognised as a sentinel event for underlying osteoporosis. Women who sustain a fragility DRF have a two- to fourfold increased risk of subsequent hip fractures within the subsequent decade [2]. Despite this well-established association, numerous studies have documented a persistent secondary prevention gap, where fewer than 20% of patients with fragility DRFs receive appropriate bone mineral density assessment or anti-osteoporotic therapy [11]. Comparatively, a Swedish national registry study reported that only 13% of elderly patients with DRFs underwent bone health assessment within one year of fracture [12]. In Asia, the situation appears even more concerning. Sebastin and Chung highlighted that across many Asian healthcare systems, including Malaysia, Singapore, and Thailand, osteoporosis screening after fragility fractures remains grossly underutilised due to resource constraints, lack of awareness, and fragmented care pathways [13]. Our data underscore the urgent need for systematic implementation of fracture liaison services in Malaysian hand and microsurgery units, as has been successfully done in parts of Europe and Australia.

The distribution of fracture types in our cohort, with OTA type A comprising 70.5% of cases, is consistent with other published series. Zugasti-Marquinez et al. reported a similar predominance of extra-articular fractures (78.4%) in a Spanish population, while Ando et al. found that type A fractures accounted for 68.7% of DRFs in their Japanese registry [7,14]. The relatively lower proportion of complex intra-articular fractures (OTA type C, 6.7%) in our study compared to some Western series may reflect differences in referral patterns, injury mechanisms, or classification practices.

Our overall conservative treatment rate of 75.4% is considerably higher than that reported in many developed countries. Viberg et al., in a 22-year Danish nationwide registry study of 276,145 fractures, documented a steady increase in surgical intervention rates from 9% to 23% over the study period. Similarly, the Swedish Fracture Register reported that 47% of all DRFs underwent operative treatment [12,15]. The lower surgical rate in our setting cannot be simply attributed to case mix, as even for OTA type C fractures, which are generally accepted as indications for surgery, only 69.2% underwent operative fixation compared to near-universal surgical treatment in most Western series. Several factors may explain this discrepancy. Gutiérrez-Espinoza et al. conducted a systematic review and meta-analysis comparing surgical versus conservative treatment in elderly patients and found that while surgery provides superior radiographic alignment, there are no clinically meaningful differences in patient-reported functional outcomes at one year [16]. This evidence supports a more selective approach to operative intervention, particularly in older, lower-demand patients. Additionally, healthcare system factors in Malaysia, including financial barriers, operating theatre access, and surgeon training patterns, likely influence treatment decisions [13]. Anil et al. similarly reported a high rate of conservative management (68.4%) in their South Indian cohort, suggesting that nonoperative treatment remains the norm across much of South and Southeast Asia [6].

Our data reveal an interesting and evolving surgical trend: the use of combined volar and dorsal plating in 3.3% of cases and isolated dorsal plating in one patient. While these numbers remain small, they represent a departure from the traditional paradigm of isolated volar plating, which has dominated DRF surgery for the past two decades. Dorsal plating for DRFs has historically been associated with higher rates of extensor tendon complications, including tenosynovitis, tendon irritation, and rupture [11]. However, contemporary low-profile dorsal plates with improved biomechanical design have reduced these complications, leading to a resurgence of interest in dorsal approaches for specific fracture patterns. Indications for dorsal or combined plating include dorsal shear fractures (Die-punch variants), fractures with significant dorsal comminution, and certain intra-articular patterns where volar alone cannot provide adequate buttress [8]. A study from the United States using the National Trauma Data Bank found that dorsal plating accounted for approximately 8% of operatively treated DRFs, with use increasing over time [8]. Our observed rate of 3.6% for combined or isolated dorsal plating, while lower than the 8% reported in US cohorts, suggests an emerging trend toward more specialised fixation in Malaysia. This shift likely reflects the increased availability of contemporary low-profile dorsal plates, which mitigate historical concerns regarding extensor tendon complications, alongside a growing institutional focus on providing specific buttress support for complex intra-articular patterns that cannot be adequately addressed via a volar-only approach.

Several limitations warrant consideration. This was a single-centre retrospective study, which introduces potential selection bias and limits generalisability to nontertiary settings. The absence of a Malaysian national fracture registry precludes calculation of population-based incidence rates. We did not collect functional outcome data, fracture union rates, or complication profiles, which would have strengthened comparisons between treatment modalities. Additionally, we did not document bone mineral density testing or anti-osteoporotic treatment initiation in fragility fracture patients, limiting our ability to comment on secondary prevention practices. Furthermore, a CT scan would be the best modality to classify the fracture, and it was not used as a CT scan is not a common radiological exam for distal end radius fracture. While we utilised standardised radiographs for classification, it is important to acknowledge that OTA classification based on plain film alone may underestimate intra-articular complexity compared to CT imaging. This diagnostic limitation might contribute to the relatively lower proportion of OTA type C fractures reported in our cohort compared to international registries that more frequently utilise advanced imaging.

## Conclusions

This study confirms a bimodal age distribution for DRFs in a Malaysian tertiary hand surgery centre, with distinct peaks at 30-40 years (young males, high-energy trauma) and 65-75 years (elderly females, fragility fractures). The finding that nearly half of all DRFs are fragility fractures represents an alarming public health challenge and an urgent call for fracture liaison services in Malaysia. Conservative management remains the dominant treatment strategy, consistent with evidence that functional outcomes may not differ substantially from surgery in elderly populations. However, emerging surgical trends, including the use of combined volar-dorsal plating for complex fracture patterns, warrant prospective evaluation. In an era of ageing populations and constrained healthcare resources, epidemiological studies such as this provide essential evidence to guide prevention, resource allocation, and treatment protocols.
